# Liraglutide Protects Nucleus Pulposus Cells Against High-Glucose Induced Apoptosis by Activating PI3K/Akt/ mTOR/Caspase-3 and PI3K/Akt/GSK3β/Caspase-3 Signaling Pathways

**DOI:** 10.3389/fmed.2021.630962

**Published:** 2021-02-19

**Authors:** Mingyan Yao, Jing Zhang, Zhihong Li, Xiaoliang Bai, Jinhui Ma, Yukun Li

**Affiliations:** ^1^Department of Endocrinology, The Third Hospital of Hebei Medical University, Shijiazhuang, China; ^2^Department of Endocrinology, Baoding No.1 Central Hospital, Baoding, China; ^3^Department of Cardiology, Affiliated Hospital of Hebei University, Baoding, China; ^4^Department of Orthopedics, Baoding No.1 Central Hospital, Baoding, China; ^5^Department of Endocrinology, Affiliated Hospital of Hebei University, Baoding, China

**Keywords:** apoptosis, liraglutide, nucleus pulposus cells, signaling pathway, diabetes mellitus

## Abstract

**Background and Objective:** Diabetes mellitus (DM) is reportedly a significant risk factor for intervertebral disc degeneration (IDD). Incretin system and particularly glucagon-like peptide 1 (GLP-1) because of its glucose-lowering effects has become an important target in therapeutic strategies of type 2 diabetes (T2D). Liraglutide is a GLP-1 receptor (GLP-1R) agonist with glucoregulatory and insulinotropic functions as well as regulatory functions on cell proliferation, differentiation, and apoptosis. However, little is known on the roles and signaling pathways of apoptosis protecting effects of liraglutide in IDD. This study aimed to investigate the potential protective effects of liraglutide against high glucose-induced apoptosis of nucleus pulposus cells (NPCs) and the possible involved signaling pathways.

**Methods:** The human NPCs were incubated with 100 nM liraglutide alone or in combination with LY294002 (PI3K inhibitor), rapamycin (mTOR inhibitor), and SB216763 (GSK3β inhibitor) in a high glucose culture for 48 h. The four groups were assessed further for apoptosis and genes expressions. The apoptotic effect was evaluated by flow cytometry and further confirmed by cell death detection enzyme-linked immunoassay plus (ELISAPLUS). The gene and protein expression levels were assessed by quantitative real-time polymerase chain reaction (qRT-PCR) and Western blotting techniques. The results were comparatively assessed between the four groups.

**Results:** The results confirmed the presence of GLP-1R in the NPCs indicating that liraglutide inhibited the high glucose-induced apoptosis, which was blocked by silencing GLP-1R with siRNA. Moreover, liraglutide stimulated the phosphorylation of Akt, mTOR and GSK3β. Treatment with LY294002 significantly increased the apoptosis of NPCs and reduced the levels of their downstream substrates (p-AKT, p-mTOR, and p-GSK3β). Further assessments revealed that activation of mTOR and GSK3β was almost completely inhibited by rapamycin and SB216763, respectively, which significantly increased the caspase-3 levels.

**Conclusion:** Liraglutide could protect NPCs against high glucose-induced apoptosis by activating the PI3K/AKT/mTOR/caspase-3 and PI3K/AKT/GSK3β/caspase-3 signaling pathways.

## Background

Intervertebral disc degeneration (IDD) is a common disease worldwide with significant socioeconomic burden and adverse effects on quality of life in patients. IDD has been reported as a significant contributor to low back and leg pain (1). Diabetes mellitus (DM) has been reportedly a significant risk factor for several disorders including IDD (2–4). Incretin system and particularly glucagon-like peptide 1 (GLP-1) because of its glucose-lowering effects has become an important target in therapeutic strategies of type 2 diabetes (T2D). The intervertebral disc (IVD) is consisted of the three distinct regions including nucleus pulposus, annulus fibrosis and cartilage endplate, providing stability and flexibility to the spinal column (5), and it is mainly caused by the decline in the quantity and activity of nucleus pulposus cells (NPCs) (6). High glucose environment has been previously reported to inhibit cellular proliferation and induce cell apoptosis, which subsequently promote the IDD progression (4, 7). Therefore, suppression of high glucose-induced aberrant apoptosis might be helpful in preventing the development of IDD in diabetic patients.

Incretin system has become an important therapeutic target for T2D treatment, mainly because of “the incretin effect,” which explains oral glucose ingestion results in greater insulin secretion, compared to isoglycemic intravenous glucose infusion (8, 9). Incretin hormones are gastrointestinal hormones produced by the intestinal mucosa after oral nutrient intake and are capable of promoting insulin secretory responses and lowering blood glucose levels in a glucose-dependent manner during hyperglycemic conditions (10–12). Incretins decrease insulin release when glucose levels are approximately normal. Two incretin hormones are identified so far including glucose-dependent insulinotropic polypeptide and GLP-1 (13). GLP-1 has drawn significant research interest mainly because of its capacities in lowering glucose, slowing gastric emptying, improving insulin sensitivity, and inhibiting glucagon secretion (14–18). Moreover, GLP-1 is actively involved in regulation of cell proliferation and apoptosis through multiple pathways (19–21). However, the therapeutic applications of native GLP-1 are limited mainly due to its rapid degradation and short half-life. GLP-1 receptor (GLP-1R) agonists with long half-life that are resistant to degradation can overcome this limitation (17, 22, 23). Liraglutide is a human GLP-1R analog that shares a 97% homology with endogenous GLP-1, with a significantly longer half-life of about 13 h. It is a powerful antidiabetic agent and has been reported to inhibit oxidative stress and apoptosis in various cells (24, 25). However, to our knowledge, there is no published study the role and molecular mechanism underlying of liraglutide in IDD.

The phosphatidylinositol 3-kinase (PI3K)/protein kinase B (Akt) signaling pathway is an intracellular signal transduction pathway involved in the regulation of multiple cellular and molecular physiological processes and plays vital roles in metabolism, proliferation, cell survival, growth, migration, angiogenesis, and apoptosis in response to extracellular signals (10, 26–29). The activation form of phosphor-Akt (p-Akt) can promote cell proliferation and inhibit cell apoptosis by regulating downstream proteins. It has been reported that GLP-1 protects insulin-secreting cells from H_2_O_2_-induced apoptosis through Cyclic adenosine monophosphate (cAMP) and PI3K dependent signaling pathways (30). Moreover, studies on C3H10T1/2 mesenchymal stem cells and MC3T3-E1 cells have demonstrated that the protective effects of GLP-1 are possibly mediated by activation of Akt process and possibly its downstream of mechanistic target of rapamycin kinase (mTOR) and Glycogen synthase kinases-3β (GSK3β) (31, 32).

In the present study we aim to investigate the potential protective effects of liraglutide, GLP-1R agonist, against high glucose-induced apoptosis in the NPCs and the possible involved signaling pathways that mediate the anti-apoptotic action of liraglutide.

## Materials and Methods

### Cell Culture and Experimental Design

All the experiments and assessments in this study were approved by local ethics committee of the Third Hospital of Hebei Medical University, Shijiazhuang, China which were in complete accordance with the ethical standards and regulations of human studies of the Helsinki declaration (2014). Human NPCs were purchased from American Science Cell Research Laboratories and cultured in Nucleus Pulposus Cell Medium (NPCM) according to the manufacturer guidelines and the method previously described (23). The cultivation process was performed under NPCM with standard conditions (37 C, 21% O_2_ and 5% CO_2_). The cultivation medium contained 500 ml of basal medium, 10 ml of Fetal Bovine Serum (FBS), 5 ml of NPC growth supplement, and 5 ml of penicillin/streptomycin solution (P/S) (Hyclone, Logan, UT, USA). The cells were washed with fresh medium at the next day to remove unattached cells and residual DMSO (Solarbio, Beijing, China), then were washed every 2–3 days. Once the NPCs reached 70% confluency, the medium was changed every other day until reaching approximate 80–90% confluency. Finally, the NPCs were split 1:3 and then subcultured using 0.25% (w/v) trypsin solution (Sigma, St.Louis, MO, USA).

The resultant third-generation of NPCs were randomly divided into six groups as follows: control (CON) group: cultured in NPCM; High-glucose (HG) group: cultured in high glucose concentration (0.2 M) medium; High-glucose plus liraglutide (HG + LIR) group: cultured in high glucose medium containing liraglutide (100 nM); High glucose plus liraglutide plus LY294002 (HG + LIR + LY) group: cultured in high glucose medium containing liraglutide (100 nM) and a phosphatidylinositol 3-kinase inhibitor (LY294002, 20 μM); High glucose plus liraglutide plus rapamycin (HG + LIR + RAPA) group: cultured in high glucose medium containing liraglutide (100 nM) and rapamycin (inhibitor of mTOR); and high glucose plus liraglutide plus SB216763 (HG + LIR + SB) group: cultured in high glucose medium containing liraglutide (100 nM) and SB216763 (an inhibitor of GSK-3β). The concentrations of agents like liraglutide and inhibitors and cultivating conditions of the experimental groups were determined according to the previously similar studies (23, 32). In all the six groups, the cell vitality, apoptosis rate, and the expression of proteins and genes were determined under the same experimental conditions after 48-h incubation interval.

### Measurement of Cell Viability

The cell viability in all the experimental groups was measured by Cell Counting Kit-8 assay (CCK-8, Sigma Aldrich, USA). The CCK-8 assay allows sensitive colorimetric assays for the determination of the number of viable cells in the proliferation and cytotoxicity assays. It uses WST-8 (2-(2-methoxy-4-nitrophenyl)-3-(4-nitrophenyl)-5-(2,4-disulfophenyl)-2H-tetrazolium, monosodium salt) that produces a water-soluble formazan dye upon bioreduction in the presence of an electron carrier, 1-Methoxy PMS. We performed the cell viability assay as per the instructions of the manufacturer and the established method described in the previous study (23). Briefly, the NPCs were seeded in 96-well culture plates at a density of 1.0 × 10^5^/ml (3 wells for each group and 100 μl in each well) in a CO_2_ incubator. When reached a confluence of ~90%, different media were replaced according to our experimental design corresponding to the different interventions and control groups. After 48 h incubation, the CCK-8 assay reagent (10 μl) was added directly to the culture medium for an additional 3 h. The WST-8 was bioreduced by cellular dehydrogenases to an orange formazan product that is soluble in the culture medium. The amount of produced formazan was directly proportional to the number of living cells. A microplate reader (Dynatech MR5000, Eggenstein, Germany) was used to measure the absorbance of each well at 450 nm.

### Apoptosis Assessment by Flow Cytometric Analysis

Cell apoptosis was quantitatively determined using an Annexin V-FITC apoptosis detection kit (BD Pharmingen, USA) with propidium iodide (PI) as the viability probe. The assay determines the percentage of cells within a population that are actively undergoing apoptosis. The PI probe was used to determine early apoptotic cells (PI negative, FITC Annexin V positive). The assay was performed as per the instructions of the manufacturer. Briefly, the NPCs were collected by centrifugation (1,000 rpm/min, 5 min, 4°C) after trypsinization with 0.25% trypsin (Sigma, St. Louis, MO, USA) and washing twice with phosphate buffer solution (PBS, Solarbio, China). In the next step, the cells were resuspended in 200 μl of binding buffer and then stained with 10 μl of FITC-Annexin V solution under dark condition for 15 min according to the manufacturer's instructions. Then, 300 μl of binding buffer and 10 μl of PI were added for another 5 min. Finally, NPCs were subjected to a flow cytometry machine (BD Biosciences, San Jose, CA, USA) to analyze the apoptotic cell ratio. The determination of cells was based as follows. The cells with both FITC Annexin V and PI negative were considered viable; cells with FITC-Annexin V positive and PI negative were in early apoptosis; and cells with both FITC-Annexin V and PI positive were counted as late apoptosis or already dead populations. Each experiment was repeated three times independently and the averaged values were used for analyses. The data were expressed as a percentage of the total cell count.

### Apoptosis Determination by ELISA

The cell death detection enzyme-linked immunoassay (ELISA) plus kit (Roche Molecular Biochemicals, Germany) was used to determine cell apoptosis. This assay qualitatively and quantitatively determined the cytoplasmic histone-associated-DNA-fragments (mono- and oligonucleosomes) after induced cell death. It was based on the quantitative sandwich-enzyme immunoassay-principle using mouse monoclonal antibodies directed against DNA and histones, respectively. The assessment was performed as per the instructions of the manufacturer. Briefly, the NPCs with different treatments were lysed for 30 min at room temperature (25°C), following by centrifugation for 10 min. The amount of the DNA fragments detected in the supernatants showed the extent of apoptosis in the sample.

### Quantitative Real-Time Polymerase Chain Reaction (qRT-PCR)

The qRT-PCR technique was used to detect and quantify the expression level of mRNA encoding Bcl-2, Bax, and caspase-3, respectively. The total RNA of NPCs was extracted using the TRIzol reagent (Invitrogen, USA) and quantified fluorometrically using a CyQuant-Cell Proliferation Assay Kit (Molecular Probes, Eugene, OR, USA), and then reverse-transcribed into cDNA using a ThermoScript RT KIT (Invitrogen, Shanghai, China) according to the manufacturer's instructions. The DNA sequence alignments and primer design for each gene were conducted using Primer Premier 5.0 software (Premier Biosoft International, Palo Alto, California, USA). The sequences of forward/ reverse primers used in this study are presented in Table 1.

**Table 1:** Real-time PCR primers [Sequences of forward (F) and reverse (R) primers] used in this study.

**Gene**	**Primer sequences**
Caspase-3	F: 5′-TGTTTCCCTGAGGTTTGCTG-3′
	R: 5′-TGCTATTGTGAGGCGGTTGT-3′
Bcl-2	F: 5′-TTCTTTGAGTTCGGTGGGGTC-3′
	R: 5′-TGCATATTTGTTTGGGGCAGG-3′
Bax	F: 5′-TCCACCAAGAAGCTGAGCGAG-3′
	R: 5′-GTCCAGCCCATGATGGTTCT-3′
β-actin	F: 5′-CAGGGCGTGATGGTGGGCA-3′
	R: 5′-CAAACATCATCTGGGTCATCTTCTC-3′

The cDNA synthesis and amplification approach was employed to determine the expression level of mRNA as per the instructions of the manufacture. A GoScript^TM^ Reverse Transcription System (Promega, USA) was used in a standard volume of 20 μl consisting 10 μl Master Mix, 0.2 μl forward primer, 0.2 μl reverse primer, 2 μl cDNA, and 7.6 μl nuclease-free water. The PCR amplification was performed with the following protocols: 95°C for 3 min, then 40 cycles of 95°C for 10 s, and finally 60°C for 30 s. Standard curves were run in each assay, which produced a linear plot of threshold cycle (Ct) against log (dilution). The expressions of the target gene were quantified based on the concentration of the standard curve and then were presented as relative Ct values. The transcription levels of β-actin were served as a loading control and the transcription levels for all six experimental groups were compared.

### Small Interfering RNA (siRNA) Silencing

Small interfering RNA (siRNA) was used to silence the GLP-1R in the NPCs. Transfection of human NPCs with siRNA of the GLP-1R was performed according to the previously described method (33, 34). Briefly, the siRNA was specific and negative siRNAs were purchased from GenePharma Biotechnology, Shanghai, China. After isolation of the NPCs, cells were incubated for 1 h in NPCM medium. The Entranster TM-R4000 (Engreen Biosystem Co, Ltd., New Zealand) was used according to the protocol of the manufacturer employing 200 nM GLP-1R siRNA. Cells were treated with negative control siRNA (SiCON) or GLP-1R siRNA (SiGLP-1R) before liraglutide (LIR) treatment in a high-glucose culture. After transfection, cells were incubated in 12-well plates with NPCM medium for 48 h. Untreated cells as well as the cells treated with negative siRNA were used as negative controls. The siRNA silencing efficiency was determined 48 h post transfection by protein analysis for further experiments.

### Western Blotting Analysis

In this study, the protein levels were detected by Western blotting technique, with β-actin as internal reference protein. For Western blotting assay, following appropriate treatment where applicable, the NPCs were homogenized using a radioimmunoprecipitation assay (RIPA) lysis buffer (Beyotime, China) on the ice for 20 min. A Protein BCA Kit (Beyotime, China) was employed to quantify the protein concentration. Then, equal amounts of protein samples were separated by 10% sodium dodecyl sulfate polyacrylamide gel electrophoresis (SDS-PAGE) and then transferred to the polyvinylidene difluoride (PVDF) membranes (Merck Millipore, Billerica, MA, USA). In the final step, the transferred PVDF membranes were blocked against tissue digestion using 5% non-fat milk in Tris Buffered Saline with Tween® 20 (TBST) solution (50 mmol/l Tris, pH: 7.6, 150 mmol/l NaCl, 0.1%) for 1 h at the room temperature (25°C) and then incubated with the appropriate concentration of primary antibodies (Proteintech, Wuhan, China) at 4°C overnight. On the second day, PVDF membranes were washed out three times in the TBST solution and then incubated with the secondary antibodies (Proteintech, Wuhan, China) for 2 h at 37°C. The immunoreactivity bands were interacted with an enhanced chemiluminescence (ECL) kit (Thermo, USA), and the gray values were analyzed using ImageJ software (National Institutes of Health, USA).

### Statistical Analysis

Statistical analyses were conducted by Statistical Package for the Social Sciences (SPSS) (Windows, version 22.0). All data were presented as Mean ± Standard Deviation (SD) from the results of at least three independent experiments. Statistical analysis among multiple groups was analyzed by the one way analysis of variance (ANOVA), and the *post-hoc* test was performed using the SNK-q test or the least significant difference (LSD) test. *P* < 0.05 was considered statistically significant.

## Results

### Liraglutide Inhibits High-Glucose Induced Apoptosis in NPCs

**Figure 1:**
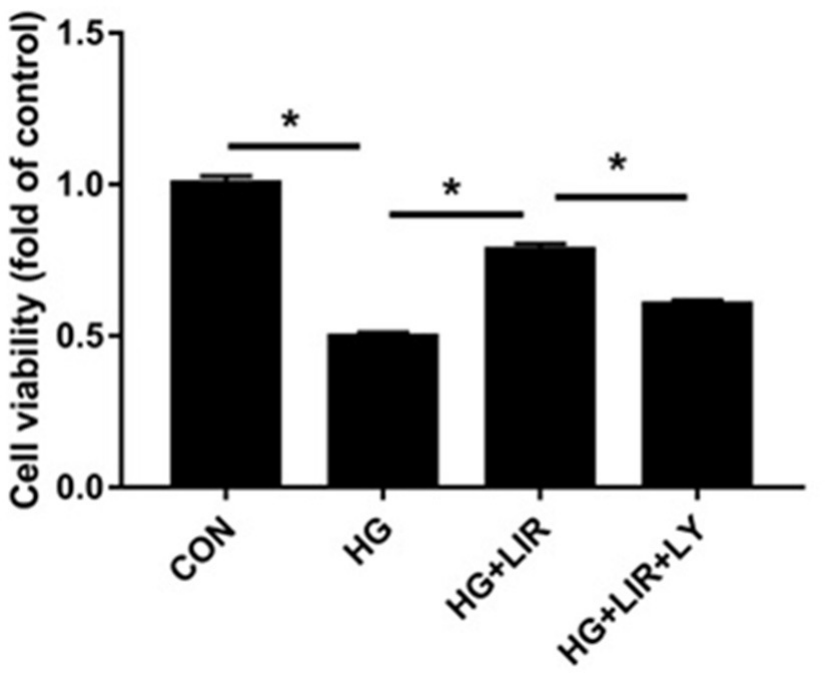
NPCs proliferation activity under different treatments. Cell Counting Kit 8 (CCK-8) assay was used to assess cell proliferation activity. NPCs were treated in four groups as presented in the figure. CON, control; HG, high glucose; LIR, liraglutide; LY, LY294002. Data are expressed as Mean ± SD (*n* = 3). *P < 0.05.

The cell proliferation activities in all experimental groups were measured using the CCK-8 assay. The results showed that the cell proliferation activity in the high-glucose group significantly decreased, compared to the control group (*P* < 0.05). The addition of liraglutide increased the proliferation activity, which was then reversed by adding the inhibitor LY294002 (Figure 1) (*P* < 0.05).

**Figure 2:**
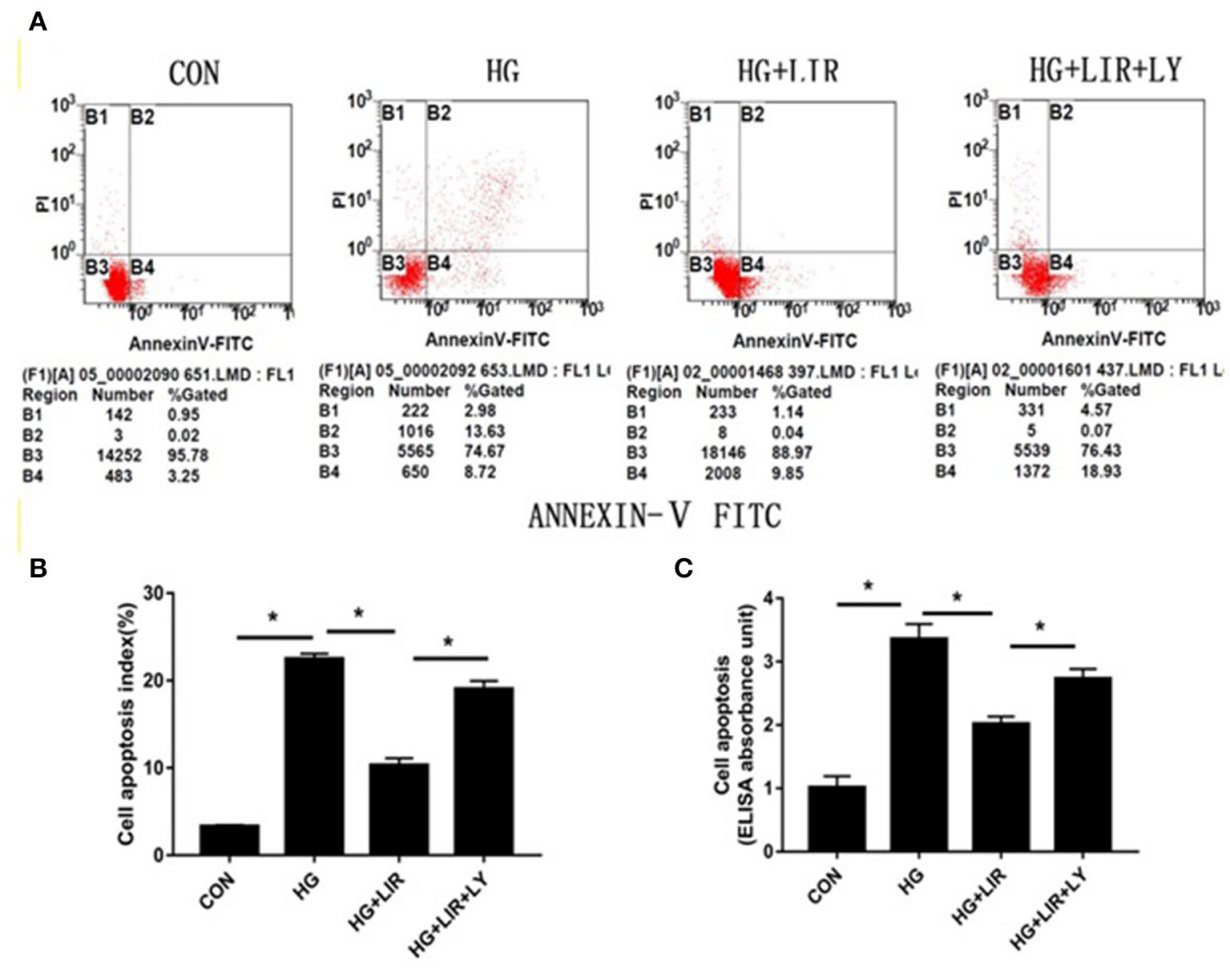
NPCs apoptosis ratio for different treatments. Apoptotic NPCs were detected and confirmed by FITC-Annexin V-PI staining **(A,B)** and Cell Death ELISAPLUS **(C)**, respectively. CON, control; HG, high glucose; LIR, liraglutide; LY, LY294002. Data are expressed as Mean ± SD (*n* = 3). *P < 0.05.

The apoptotic effect on the NPCs was evaluated and confirmed by FITC-Annexin V-PI staining and Cell Death ELISAPLUS assessments, respectively. The results showed that high-glucose treatment (0.2 M) significantly increased the percentage of apoptosis, compared with the control group (*P* < 0.05). Moreover, addition of liraglutide suppressed the NPC apoptosis, whereas inhibition of the PI3K/Akt pathway by LY294002 counteracted the inhibiting effects of liraglutide in the high-glucose group (*P* < 0.05) (Figure 2).

### Liraglutide Regulates the Apoptosis Related Gene Expressions in NPCs

**Figure 3:**
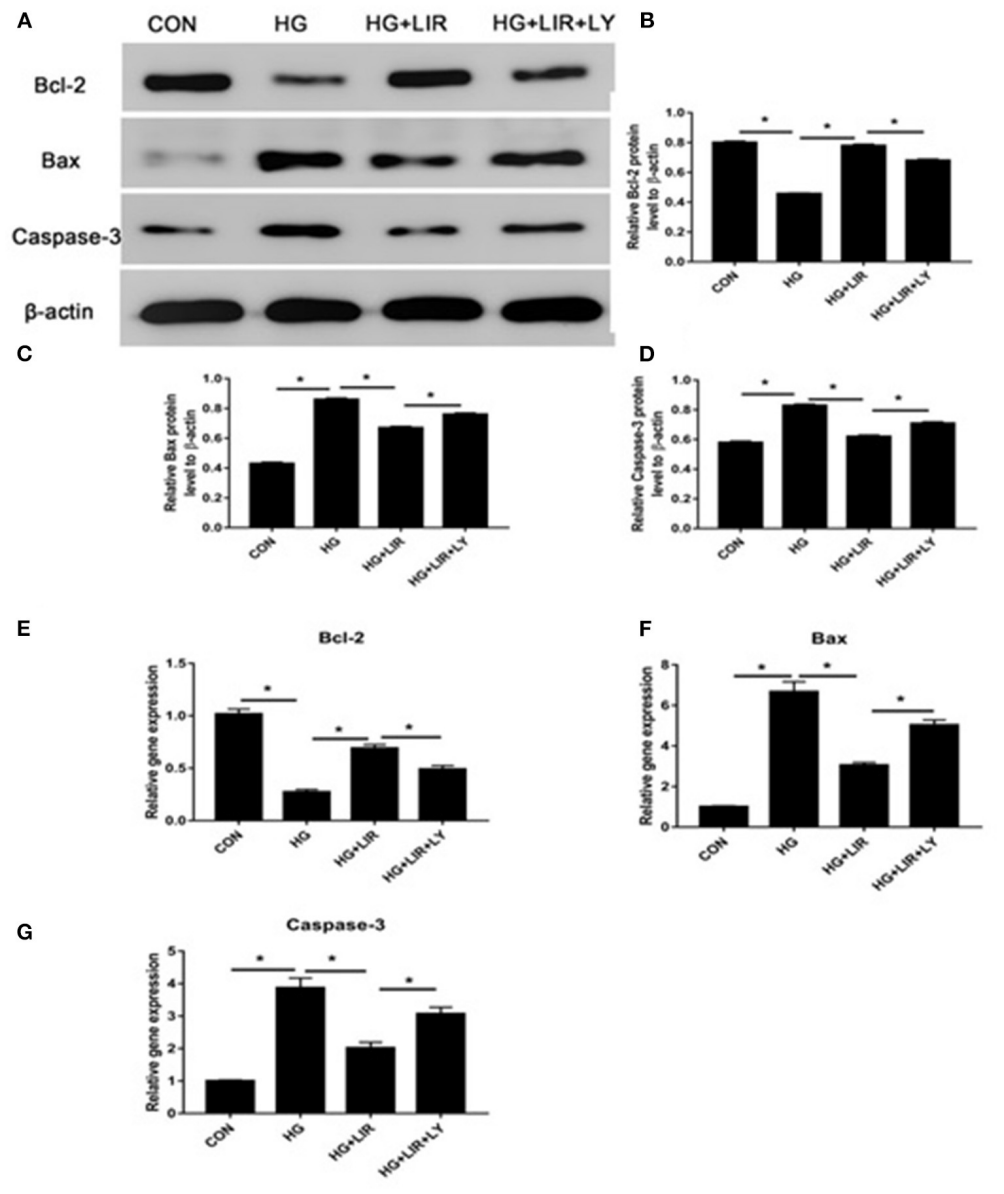
The expressions of pro- and anti-apoptosis molecules in the different treatment groups using Western blot and RT-PCR techniques. The expressions of Bcl-2, Bax, and caspase-3 by Western blot **(A–D)** and RT-PCR **(E–G)**. Liraglutide partly decreased the expressions of Bax and caspase-3, whereas increased the Bcl-2 expression in a high glucose culture and the inhibitor LY294002 partly reversed the liraglutide-induced effects. CON, control; HG, high glucose; LIR, liraglutide; LY, LY294002. Data are presented as Mean ± SD (*n* = 3). *P < 0.05.

Our results showed that the high-glucose treatment significantly upregulated the expression of pro-apoptotic molecules (Bax and caspase-3), whereas down-regulated the anti-apoptotic molecule (Bcl-2) (*P* < 0.05). Liraglutide partly decreased expression of the pro-apoptotic molecules (Bax and caspase-3), whereas increased expression of the anti-apoptotic molecule (Bcl-2) in a high glucose medium, and the LY294002, a PI3K inhibitor, partly reversed the liraglutide-induced effects of in the high glucose group (*P* < 0.05) (Figure 3).

### PI3K/Akt/mTOR/Caspase-3 and PI3K/Akt/GSK3β/Caspase-3 Pathways Mediate Liraglutide Anti-apoptosis Effects in NPCs

**Figure 4:**
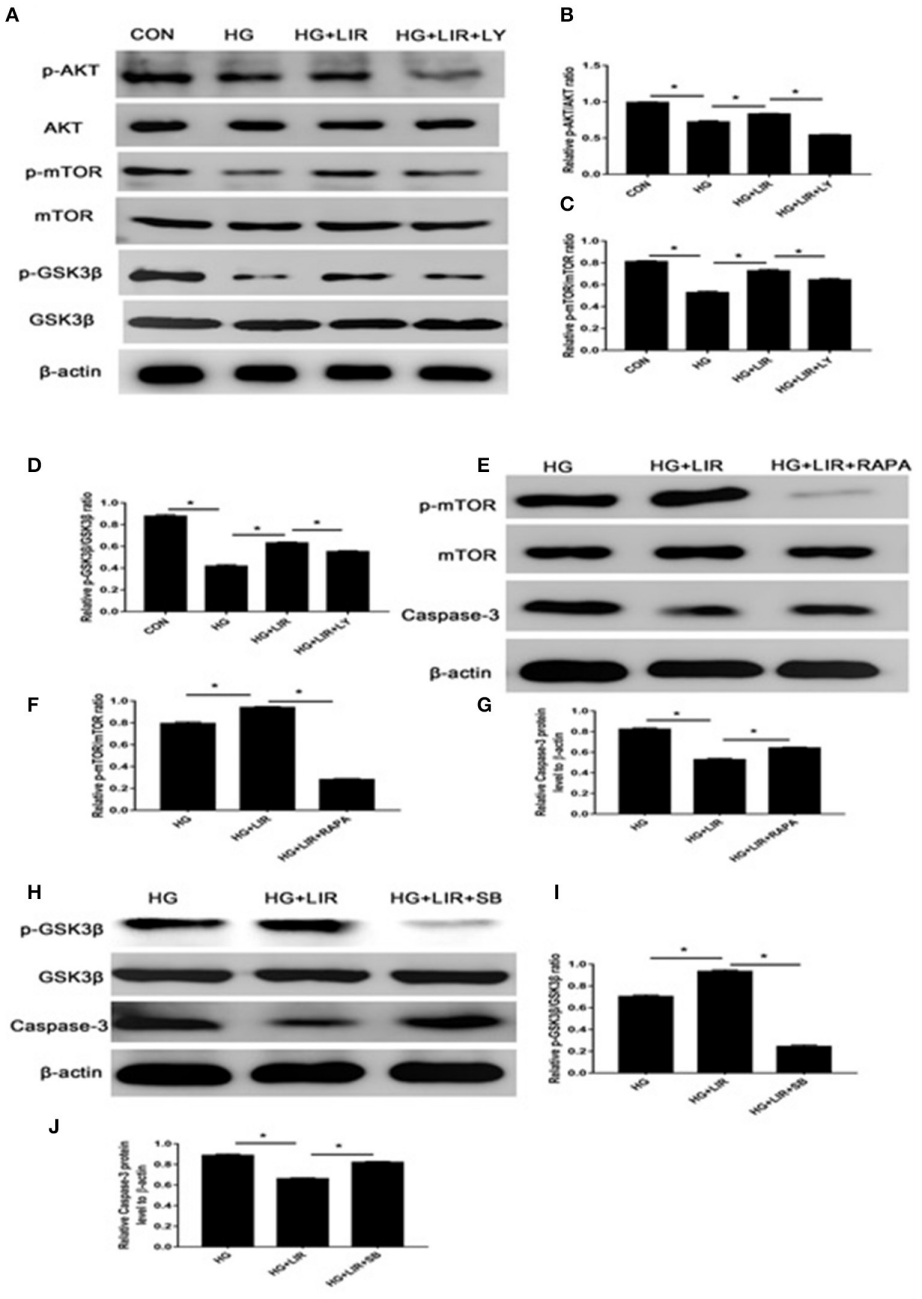
The Western blot assessments of the activations of PI3K/Akt/mTOR/caspase-3 and PI3K/Akt/GSK3β/caspase-3 signaling pathways in anti-apoptotic effects of liraglutide. Cells were exposed to liraglutide (100 nM) and LY294002 (20 μM), respectively. The outcomes of Western blotting on the expressions of Akt **(A,B)**, mTOR **(A,C)**, and GSK3β **(A,D)**. In further assessments, cells were incubated with 100 nM liraglutide and rapamycin or SB216763, respectively. Results of Western blot assessments on expressions of mTOR **(E,F)**, GSK3β **(H,I)**, and caspase-3 **(G,J)**. CON, control; HG, high glucose; LIR, liraglutide; LY, LY294002. Data are expressed as Mean ± SD (*n* = 3). *P < 0.05.

The activation status and the triggering factors of the intracellular signaling pathways were investigated to determine the molecular mechanisms involved in exerting the anti-apoptosis effects of liraglutide in NPCs. Our results showed that high-glucose treatment of NPCs significantly reduced the expression of anti-apoptotic proteins p-Akt, p-mTOR, and p-GSK3β in the cell apoptosis. Contrary, the liraglutide treatment significantly promoted the phosphorylation of Akt, mTOR and GSK3β, compared with the high-glucose group (*P* < 0.05) (Figures 4A–D). The high-glucose treatment significantly upregulated the level of pro-apoptotic caspase-3 protein, whereas liraglutide addition to the medium markedly reduced the expression level of this protein (Figures 3A,D). As it was expected, the inhibitor LY294002 significantly decreased the levels of p-Akt, p-mTOR, and p-GSK3β expressions, which liraglutide addition significantly increased the levels of caspase-3 in a high glucose culture (*P* < 0.05) (Figures 4A–D).

To further evaluate the apoptosis-inhibiting effect of liraglutide and the role of mTOR and GSK3β as the downstream substrates of Akt process in the NPCs under high-glucose medium, additional Western blot assessments were performed. The cells were incubated with rapamycin (inhibitor of mTOR) and SB216763 (inhibitor of GSK3β) in the high glucose medium combined with liraglutide group. The rapamycin and SB216763 completely prevented the activation of mTOR and GSK3β, respectively (*P* < 0.05). Moreover, rapamycin and SB216763 treatments significantly increased the caspase-3 expression levels in the NPCs (*P* < 0.05) (Figures 4E–J). These findings demonstrated that liraglutide could stimulate the PI3K/Akt/mTOR/caspase-3 and PI3K/Akt/GSK3β/caspase-3 signal transduction pathways in the NPCs under high-glucose environment.

### Role of GLP-1R in Liraglutide Induced NPCs Apoptosis

**Figure 5:**
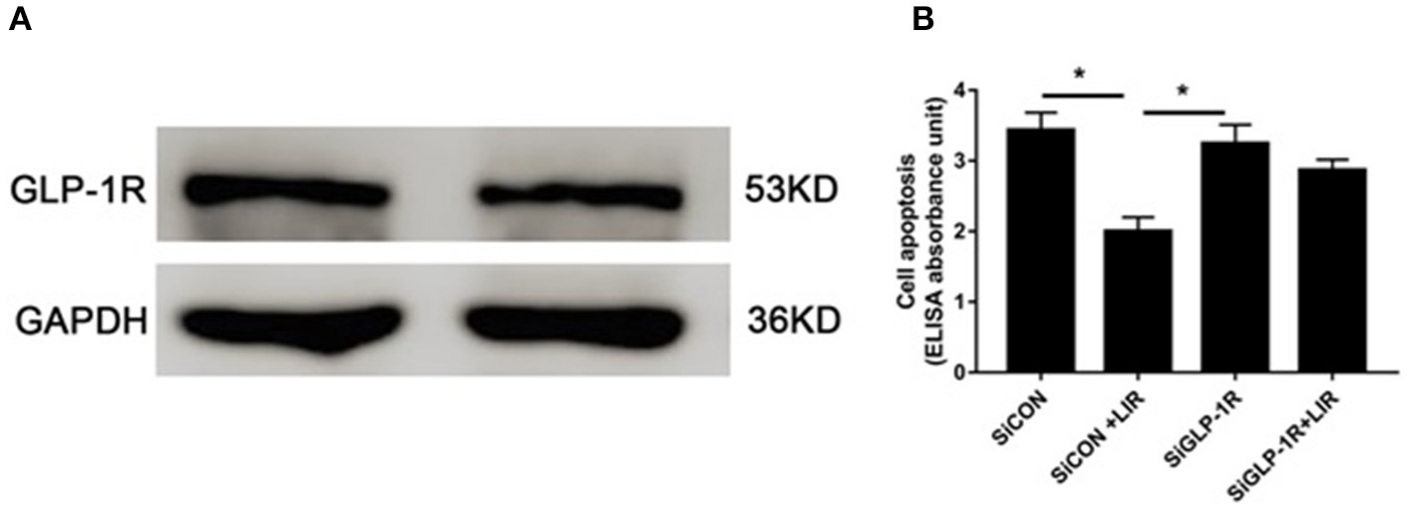
Role of GLP-1R in NPCs apoptosis under high-glucose treatment. **(A)** Western blot analysis showed GLP-1R protein was expressed in the NPCs. **(B)** Silencing GLP-1R with siRNA transfection lead to the liraglutide anti-apoptotic effect inhibition. Before liraglutide (LIR) treatment, cells were treated with GLP-1R siRNA (SiGLP-1R) or negative control siRNA (SiCON), then assessed for apoptosis using cell death detection ELISAPLUS assay. Data are presented as Mean ± SD (*n* = 3). *P < 0.05.

The Western blot assessments demonstrated the presence of GLP-1R protein in the NPCs (Figure 5A). Cell death ELISAPLUS was performed to determine the apoptosis of NPCs. Our data revealed that silencing GLP-1R with siRNA partly blocked the anti-apoptotic effect of liraglutide (Figure 5B), demonstrating that liraglutide prevented apoptosis of NPCs via GLP-1R.

## Discussion

This study investigated the anti-apoptotic effects of liraglutide and possible underlying molecular mechanisms in NPCs in high glucose environment. Our findings showed that the inhibition of apoptosis by liraglutide is mediated, at least in part, through PI3K/Akt/mTOR/caspase-3 and PI3K/Akt/GSK3β/caspase-3 signaling pathways. The results also revealed that GLP-1R is involved in NPCs apoptosis and liraglutide mediated inhibition of apoptosis is GLP-1R dependent.

IDD is reportedly a major risk factor for low back pain as well as chronic pain in lower extremities (1). Research is ongoing to understand the pathogenesis of disc degeneration and develop effective therapies for this disorder (35–37). In the recent years, some basic and epidemiological studies have demonstrated that DM is a potential etiological factor of IDD (38, 39). High glucose environment can significantly affect disc biology from disc cell viability to disc matrix metabolism, resulting disc NPCs apoptosis (7, 40). Therefore, inhibiting high glucose-induced NPCs apoptosis has important significance in retarding disc degeneration.

Apoptosis or programmed death is a complicated process that contributes to the structural and functional development of various multicellular organisms. This process contributes to the pathogenesis of several diseases including neurodegenerative diseases, cancer, and immune system dysfunctions. Many factors, majority of them proteins, are actively involved in the apoptosis, the most important of them are caspases, amyloid-B peptide, Bcl-2 family of proteins, p53 gene, and the heat shock proteins (41–44). Apoptotic mechanism consists of three main parts of initiation, execution, and termination. Apoptosis could be triggered by several factors such as alkylating agents, chemotherapeutic agents, oxidative stress, and ionizing radiation or by external factors such as tumor necrosis factor (TNF), cytokines, Fas ligand (FasL) and the TNF-related apoptosis inducing ligand (TRAIL) (45).

Apoptosis is activated by two distinct pathways including intrinsic and external signaling pathways. The intrinsic pathway is activated by internal signals such as DNA damage or growth factor deprivation and is regulated by protein Bcl-2. The external signal of apoptosis induction such as death activators usually bind to receptors at the cell surface.

Liraglutide, a GLP-1 agonist with long half-life, is currently employed as an interesting therapeutic agent for DM. Liraglutide, through binding to GLP-1R, exerts glucoregulatory and insulinotropic functions as well as regulatory functions on cell proliferation, differentiation, and apoptosis (46, 47). However, little is known on the functions of liraglutide in NPCs apoptosis and involved signaling pathways.

A considerable research attention has been devoted on the use of GLP-1 for treatment of T2D (8, 12, 15, 23, 48, 49). This gut incretin hormone has been reported to exert various beneficial effects on different cells including neuronal cells, pancreatic β-cells, gut and hypothalamus. These effects include lowering glucose, slowing gastric emptying, improving insulin sensitivity, enhancing glucose-dependent insulin secretory response, inhibiting glucagon secretion, inhibiting β-cell apoptosis and promoting β-cell proliferation (9, 30, 50–53). Liraglutide is a human incretin-GLP-1 agonist with 97% sequence identity identical to the native human GLP-1 but with a very longer half-life. Recent studies have reported that liraglutide could significantly prevent (by 50%) both the cytokine- and free fatty acid-induced apoptosis in primary rat islet cells in a dose-dependent manner (54).

Ming-yan et al. investigated the effects and possible mechanism of liraglutide on apoptosis of human NPCs (23). They observed that NPCs contained GLP-1R indicating that liraglutide inhibited the high glucose-induced apoptosis of NPCs. Moreover, liraglutide reduced the expression of caspase-3 activity and inhibited reactive oxygen species (ROS) generation and triggered the phosphorylation of Akt under high glucose condition. They showed that pretreatment of NPCs with a PI3K inhibitor (LY294002) prevented the anti-apoptotic effect of liraglutide on NPCs. They concluded that GLP-1R prevented the liraglutide-induced activation of Akt (23). Their findings demonstrated that liraglutide could directly protect NPCs against high glucose-induced apoptosis through impeding oxidative stress and activating the PI3K/Akt/caspase-3 signaling pathway by GLP-1R (23). In light of these findings, we designed this study to investigate the downstream substrates of Akt process in inducing protective effects against high glucose induced apoptosis deaths in NPCs through assessing the roles of mTOR and GSK3β deaths.

In our study, we chose the concentration of liraglutide at 100 nM as an optimum concentration (35, 46). Results showed that high glucose markedly increased the percentage of apoptosis and regulated the gene and protein expression related to apoptosis that down-regulated levels of anti-apoptosis molecules (Bcl-2) and up-regulated levels of pro-apoptosis molecules (Bax, caspase-3). The addition of liraglutide significantly decreased the cell apoptosis ratio, up-regulated expression of Bcl-2, and down-regulated Bax and caspase-3 in a high glucose culture.

We further explored the signaling transduction pathway in the protective effects of liraglutide against high glucose-induced NPCs apoptosis. GLP-1 has been reported to be involved in inhibition apoptosis in various cells. GLP-1 binding to GLP-1R leads to the activation of specific signaling pathways including mitogen-activated protein kinase (MAPK, phospholipase C, cAMP/PKA, and PI3K)/Akt (55). Among them, PI3K/Akt pathway plays an important role in regulating the biology of disc cells, and has been reported to interfere with the process for NPCs apoptosis (56–58). The mTOR and GSK3β are downstream effectors of the PI3K/Akt signaling, which can be activated via PI3K/Akt signaling and has been reported to inhibit apoptosis (58–61). To our knowledge, as a GLP-1 agonist, liraglutide exerts its physiological functions through binding to GLP-1R and can activate PI3K/Akt signaling cascade (35, 62). Our data show that liraglutide (100 nM) largely rescued the phosphorylation of Akt, mTOR, and GSK3β (which was inhibited by high glucose) and decreased caspase-3 levels. The LY294002, a PI3K inhibitor, blocks PI3K/Akt signaling pathway in mammalian cells. Treatment with LY294002 significantly increased the apoptosis of NPCs and reduced the levels of their downstream substrates (p-AKT, p-mTOR, and p-GSK3β). To further investigate this issue, rapamycin and SB216763 were used to inhibit activation of mTOR and GSK3β, respectively. Treatment of NPCs with rapamycin and SB216763 induced a significantly increase in caspase-3 levels, which is a critical enzyme for cell survival and apoptosis (63). Therefore, we speculated that the PI3K/Akt/mTOR/caspase-3 and PI3K/Akt/GSK3β/ caspase-3 signaling pathways are involved in the protective effects of liraglutide against high glucose-induced NPCs apoptosis. Furthermore, we ascertained that GLP-1R was expressed in the NPCs. The anti-apoptosis effect of liraglutide was blocked by the inhibition of GLP-1R with siRNA, indicating that the protective effect against high glucose-induced NPCs apoptosis is mediated by GLP-1R.

## Conclusion

This study investigated the protective effects of liraglutide against high glucose-induced apoptosis in NPCs and also the involved molecular signaling pathways. In conclusion, the present study suggested that liraglutide could attenuate high glucose induced NPCs apoptosis and the activation of PI3K/Akt/mTOR/caspase-3 and PI3K/Akt/GSK3β/caspase-3 signaling pathways may be responsible for its protective effects by binding to GLR-1R. This study provides some theoretical basis for the application of liraglutide in retarding disc degeneration, which still requires further clarification. Future studies should be conducted to determine the precise mechanisms underlying the differential effects of liraglutide on apoptosis and the related processes such as autophagy to optimize the anti-apoptotic and cytoprotective effects of liraglutide.

## Data Availability Statement

The datasets presented in this study can be found in online repositories. The names of the repository/repositories and accession number(s) can be found in the article/supplementary material.

## Ethics Statement

The studies involving human participants were reviewed and approved by local ethics committee of the Third Hospital of Hebei Medical University, Shijiazhuang, China (2018-015-1). Written informed consent for participation was not required for this study in accordance with the national legislation and the institutional requirements.

## Author Contributions

YL and MY: conceptualization. XB: methodology. JZ: software and data curation. ZL and MY: validation, writing—original draft preparation, and supervision. JM: formal analysis and resources. ZL: investigation and visualization. YL: writing—review and editing, project administration, and funding acquisition. All authors contributed to the article and approved the submitted version.

## Conflict of Interest

The authors declare that the research was conducted in the absence of any commercial or financial relationships that could be construed as a potential conflict of interest.

## Abbreviations

IDD: Intervertebral Disc Degeneration
NPCs: Nucleus Pulposus Cells
qRT-PCR: Quantitative real-time Polymerase Chain Reaction
GLP-1R: GLP-1 Receptor
IVD: Intervertebral Disc
GLP-1: Glucagon-like Peptide-1
p-Akt: Phosphor-Akt
NPCM: Nucleus Pulposus Cell Medium
FBS: Fetal Bovine Serum
Ct: Threshold Cycle
RIPA: Radioimmunoprecipitation Assay
SDS-PAGE: Sodium Dodecyl Sulfate Polyacrylamide Gel Electrophoresis
PVDF: Polyvinylidene Difluoride
TBST: Tris Buffered Saline/tween 20
ECL: Enhanced Chemiluminescence
LSD: Least Significant Difference
MAPK: Mitogen-activated Protein Kinase
PI3K: Phosphatidylinositol 3-kinase.
